# Improving the stability of ^11^C–labeled L-methionine with ascorbate

**DOI:** 10.1186/s41181-017-0032-x

**Published:** 2017-10-04

**Authors:** Michael Woods, Leo Leung, Kari Frantzen, Jennifer G. Garrick, Zhengxing Zhang, Chengcheng Zhang, Wade English, Don Wilson, François Bénard, Kuo-Shyan Lin

**Affiliations:** 1 Department of Functional Imaging, BC Cancer Agency, Vancouver, BC Canada; 2 Department of Molecular Oncology, BC Cancer Agency, Vancouver, BC Canada; 3 Department of Radiology, University of British Columbia, Vancouver, BC Canada

**Keywords:** C-11 L-methionine, Stability, Homocysteine, Methionine sulfoxide, Oxidation, Ascorbate, Positron emission tomography

## Abstract

**Background:**

Carbon-11 labeled L-methionine (^11^C–MET) is a popular tracer used in the clinic for imaging brain tumors with positron emission tomography. However, the stability of ^11^C–MET in its final formulation is not well documented in literature. Recently, we observed fast degradation of HPLC-purified ^11^C–MET over time, and systematic investigation was conducted to identify the cause.

**Results:**

In this study, we verified the degraded product as ^11^C–labeled methionine sulfoxide (^11^C–METSO). To minimize oxidation, ascorbate (100 ppm) was added to the HPLC eluant, and the resulting HPLC-purified ^11^C–MET was stable in the final formulation solution without noticeable degradation for up to 1 h after the end of synthesis.

**Conclusions:**

Our data suggest that to minimize degradation, ascorbate can be added to the ^11^C–MET formulation solution especially if it is not administered into patients soon after the end of synthesis.

**Electronic supplementary material:**

The online version of this article (10.1186/s41181-017-0032-x) contains supplementary material, which is available to authorized users.

## Background

Carbon-11 labeled L-methionine (^11^C–MET) is a promising tracer for imaging brain tumors with positron emission tomography (PET) (Watanabe et al. 2016; Maffione et al. 2009; Glaudemans et al. 2013). Its synthesis has been previously optimized by ^11^C–methylation of L-homocysteine in solution or on a C-18 Sep-Pak cartridge with or without HPLC purification (Pascali et al. 1999; Tang et al. 2004; Lodia et al. 2007; Lodi et al. 2008; Boschi et al. 2009; Cheung et al. 2009; Quincoces et al. 2010; Pascali et al. 2011; Bogni et al. 2003; Nagren et al. 1998; Oh et al. 1998; Fukumura et al. 2004). While most groups reported > 97% radiochemical purity of ^11^C–MET at the end of synthesis (EOS) even without HPLC purification, the stability of ^11^C–MET in the final formulation solution has not been well documented in literature (Pascali et al. 1999; Tang et al. 2004; Lodia et al. 2007; Lodi et al. 2008; Boschi et al. 2009; Cheung et al. 2009; Quincoces et al. 2010; Pascali et al. 2011; Bogni et al. 2003; Nagren et al. 1998; Oh et al. 1998).

**Fig. 1:**
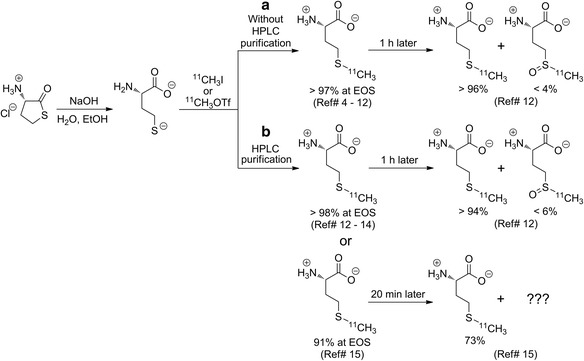
Reported radiochemical purity at the end of synthesis and stability of ^11^C–MET prepared without **a** or with **b** HPLC purification

Bogni et al. reported the formation of a minor amount (< 6%) of C-11 L-methionine sulfoxide (^11^C–METSO) at 1 h after EOS in ^11^C–MET solution prepared either with or without HPLC purification (Fig. 1) (Bogni et al. 2003). The degradation of ^11^C–MET was attributed to radiolysis as the degradation rate was affected by total radioactivity and chemical composition (ethanol and L-homocysteine) of the ^11^C–MET solutions. Fukumura et al. observed fast degradation of HPLC-purified ^11^C–MET solution with radiochemical purity at 91.2 and 72.9% for samples assayed at EOS and 20 min after EOS, respectively (Fig. 1b) (Fukumura et al. 2004). The instability of ^11^C–MET was likely caused by radiolysis as samples with higher specific activity also showed significantly faster degradation rates. The degraded radioactive product was not identified in this report. However, it was shown that adding ethanol (EtOH, 1.5%) and Tween 80 (3%) into the final formulation solution (in saline) or using saline containing ascorbate (1000 ppm) as the eluant improved the radiochemical purity of HPLC-purified ^11^C–MET to 99.9%, and no significant degradation was observed over 1 h after EOS (Fukumura et al. 2004).

In this methodology article, we communicate our experience on the preparation of HPLC-purified ^11^C–MET. We report here the instability of HPLC-purified ^11^C–MET, our systematic investigation to find out the cause of rapid degradation, and the strategy to improve the stability of ^11^C–MET in the final formulation solution.

## Results and discussion

To set up ^11^C–MET production at our institution, we used the simplest method, Sep-Pak cartridge without HPLC purification (Gomzina et al. 2011), for our initial attempt. However, we obtained much lower radiochemical purity (< 90%, data not shown) as compared to > 97% reported by others (Pascali et al. 1999; Tang et al. 2004; Lodia et al. 2007; Lodi et al. 2008; Boschi et al. 2009; Cheung et al. 2009; Quincoces et al. 2010; Pascali et al. 2011; Bogni et al. 2003). For the preparation of ^11^C–MET using Sep-Pak cartridge without HPLC purification, L-homocysteine and unhydrolyzed L-homocysteine thiolactone end up in the final formulation solution as well. In order to achieve higher chemical and radiochemical purities, we decided to use HPLC purification for subsequent preparations of ^11^C–MET.

**Fig. 2:**
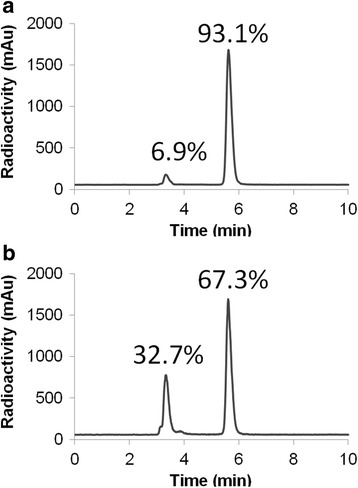
Representative radio-HPLC chromatograms of ^11^C–MET (*t*_R_ = ~5.6 min) purified by HPLC using PBS as the eluant: **a** assayed at EOS and **b** assayed at 1 h after EOS

Using phosphate-buffered saline (PBS, 3.93 mM, 3.0 mL/min) as the HPLC eluant and a C18 column (Luna C18 semi-preparative column, 5 μ, 250 × 10 mm) ^11^C–MET was obtained in 19 ± 4% (*n* = 4) decay-corrected radiochemical yield from ^11^CH_3_I with 93.5 ± 0.6% (*n* = 4) radiochemical purity and 21.0 ± 5.1 GBq/μmol specific activity at the end of synthesis (EOS). The analytical HPLC column was a Luna C18 column (5 μ, 250 × 4.6 mm), the eluant was phosphate buffer (1 mM, pH 3), and the flow rate was 1.0 mL/min. A minor radioactive by-product was observed at *t*_R_ = ~3.3 min (Fig. 2a). No residual L-homocysteine or L-homocysteine thiolactone was detected in the final ^11^C–MET solution as monitored by UV detector (set at 220 nm). The radiochemical purity of ^11^C–MET quickly dropped to 75 ± 6% (*n* = 4) at 1 h after EOS with concurrent increase of the radioactive by-product (Fig. 2b). Our radiochemical purity data (93.5% at EOS and 75% at 1 h after EOS) were lower than the data (> 99% at EOS and > 96% at 1 h after EOS) reported by Bogni et al. (Bogni et al. 2003), but were comparable to the data (91.2% at EOS and 72.9% at 20 min after EOS) reported by Fukumura et al. (Fukumura et al. 2004).

**Fig. 3:**
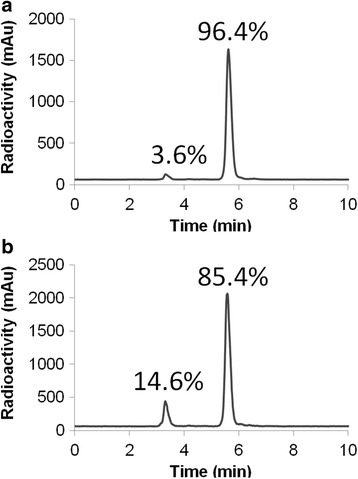
Representative Radio-HPLC chromatograms of ^11^C–MET purified by HPLC using PBS as the eluant and spiked with homocysteine: **a** assayed at EOS and **b** assayed at 1 h after EOS

To our knowledge, there was only one report on degradation of ^11^C–MET prepared using Sep-Pak cartridge without HPLC purification (Bogni et al. 2003). A minor amount (< 4%) of ^11^C–METSO was formed over a 1-h period after EOS (Bogni et al. 2003). Presumably, the remaining L-homocysteine ending up in the final formulation solution could serve as a free radical scavenger, and prevent degradation of ^11^C–MET. To verify this hypothesis, we added 0.5 mg of L-homocysteine in the product collection vial of the synthesis module. After mixing with the HPLC-purified ^11^C–MET eluate fraction, the mixed solution was passed through a sterile filter and checked by HPLC. As shown in Fig. 3a, the radiochemical purity of ^11^C–MET increased to 97.5 ± 1.5% (*n* = 2) at EOS with a minor radioactive by-product eluted at the same retention (*t*_R_ = ~3.3 min) of the degraded product as shown in Fig. 2b. However, the content of this radioactive by-product still slowly increased over time even in the presence of L-homocysteine (radiochemical purity of ^11^C–MET: 91.1 ± 8.3% at 1 h after EOS, Fig. 3b).

**Fig. 4:**
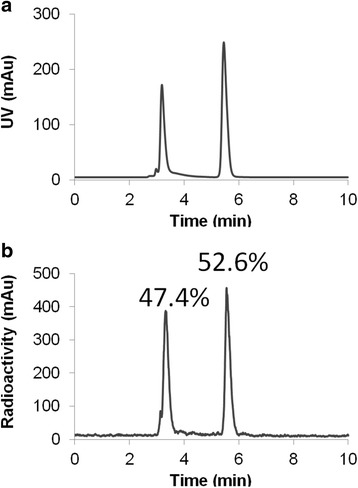
HPLC chromatograms of co-injecting MET and METSO with ^11^C–MET solution purified by HPLC using PBS as the eluant: **a** UV chromatogram **b** Radio chromatogram

Next, we tried to identify the radioactive degradation product in our HPLC-purified ^11^C–MET solution. Previously, ^11^C–METSO was reported to be the degradation product of ^11^C–MET prepared with or without HPLC purification (Bogni et al. 2003). To verify this, we co-injected the degraded ^11^C–MET solution (1 h after EOS) with MET and METSO into HPLC. As shown in Fig. 4, the UV peak of METSO co-eluted with the radio peak of the radioactive by-product (*t*_R_ = ~3.3 min), confirming ^11^C–METSO was indeed the degradation by-product of our HPLC-purified ^11^C–MET. In addition, we also conducted mass analysis for the decayed product solution, and confirmed the presence of METSO (see Additional file 1: Figure S1).

**Fig. 5:**
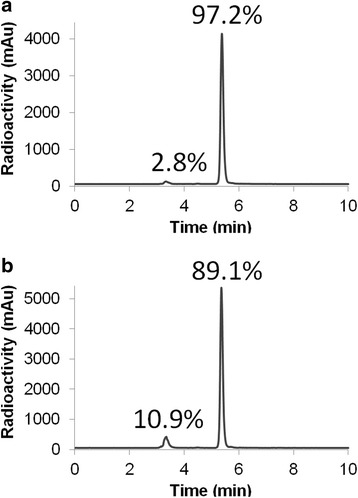
Radio-HPLC chromatograms of ^11^C–MET purified by HPLC using PBS as the eluant and spiked with EtOH (4%): **a** assayed at EOS and **b** assayed at 1 h after EOS

Previously, Bogni et al. suggested that ^11^C–MET prepared without HPLC purification could be stabilized by the remaining EtOH and L-homocysteine in the final product solution (Bogni et al. 2003). Fukumura et al. also demonstrated that HPLC-purified ^11^C–MET could be stabilized by the addition of EtOH (1.5%) and Tween 80 (3%) to the final product solution (Fukumura et al. 2004). Since EtOH is relatively nontoxic and readily available, we tested if EtOH alone could be used to stabilize the HPLC-purified ^11^C–MET solution. We added EtOH (4%) immediately after the ^11^C–MET-containing HPLC eluate fraction was collected in the final product vial, and checked radiochemical purity of ^11^C–MET over time. The radiochemical purities of EtOH-containing ^11^C–MET solution were 94.7 ± 3.3% and 77.7 ± 16.1% (*n* = 2) at EOS and 1 h after EOS, respectively. Representative HPLC chromatograms are shown in Fig. 5. These data suggest that EtOH alone is not very effective to prevent degradation of HPLC-purified ^11^C–MET solution. The previous findings by others might be due to the combination of EtOH with L-homocysteine or Tween 80 (Bogni et al. 2003; Fukumura et al. 2004).

**Fig. 6:**
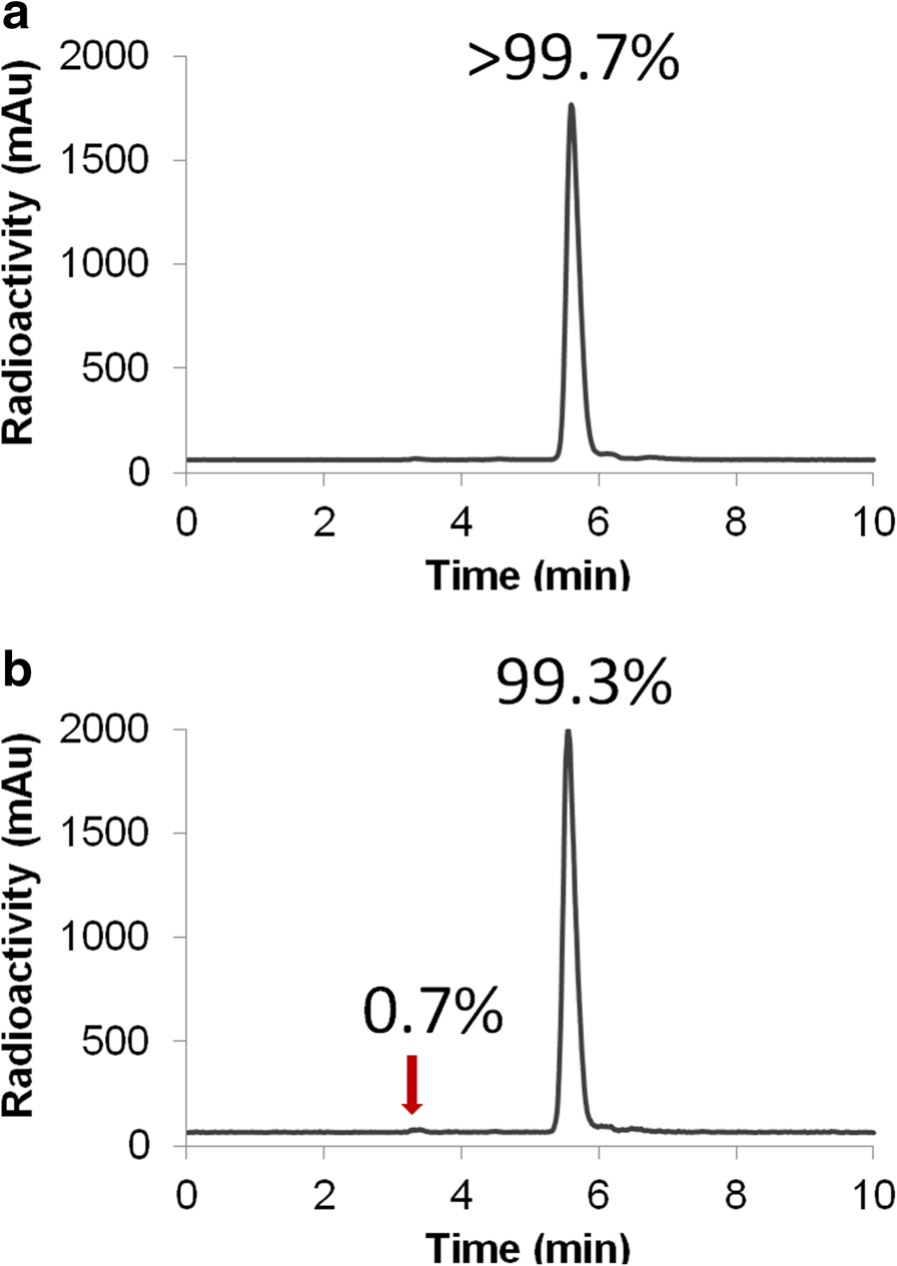
Representative radio-HPLC chromatograms of ^11^C–MET purified by HPLC using PBS containing 100 ppm of ascorbate as the eluant: **a** assayed at EOS and **b** assayed at 1 h after EOS

Interestingly, ^11^C–METSO was observed in Figs. 2, 3 and 5 even at EOS, suggesting ^11^C–METSO was formed quickly after ^11^C–MET was separated from L-homocysteine during HPLC purification. To stabilize ^11^C–MET final formulation solution and minimize the formation of ^11^C–METSO even during the HPLC purification process, we added ascorbate directly into HPLC solvent. Instead of 1000 ppm tested by Fukumura et al. (Fukumura et al. 2004), we used only 100 ppm of ascorbate. The eluate fraction containing ^11^C–MET was collected and passed through a sterile filter. ^11^C–MET was obtained in 22 ± 3% (*n* = 8) decay-corrected radiochemical yield from ^11^CH_3_I with a 99.2 ± 0.9% (*n* = 8) radiochemical purity at EOS. No significant degradation of ^11^C–MET solution was observed as the radiochemical purity was 98.2 ± 1.7% (*n* = 8) at 1 h after EOS (Fig. 6). These data are consistent with the observation of Fukumura et al. (Fukumura et al. 2004), and suggest that 100 ppm of ascorbate is sufficient to minimize the formation of ^11^C–METSO.

## Conclusions

We successfully verified the degradation of HPLC-purified ^11^C–MET was due to the formation of ^11^C–METSO. Presence of L-homocysteine or EtOH in the final ^11^C–MET formulation solution could slow down the degradation of ^11^C–MET. Adding ascorbate to the HPLC solvent greatly improved the radiochemical purity and stability of HPLC-purified ^11^C–MET solution. This could be very useful especially if ^11^C–MET is not used immediately after EOS. The tested concentration (100 ppm) contains only ~1.4 mg of ascorbate in the entire dose (~13.5 mL). It is safe for administration as this mass of ascorbate is much lower than the usual therapeutic parenteral dose (100–250 mg).

## Methods

### General methods

METSO and sodium phosphate monobasic were purchased from Sigma-Aldrich (Oakville, Canada). For preparation of the QC HPLC solvent and the phosphate buffer used to elute the reaction mixture off the cartridge, sodium phosphate monobasic was diluted with water to the specified concentrations without adjusting pH. Vitamin C injection (ascorbic acid, 250 mg/mL) and sodium phosphate injection (3 mmol/mL) were purchased from Sandoz (Boucherville, Canada). Saline injection (0.9% NaCl) was purchased from Baxter (Mississauga, Canada). The semi-preparative HPLC solvents were prepared by mixing sodium phosphate injection and saline injection (with or without vitamin C injection) to the specified concentrations. All other chemicals and solvents were obtained from commercial sources, and used without further purification. Sep-Pak tC18 Plus Short cartridges (400 mg) were obtained from Waters (Milford, MA). C-11 methane was produced by 18-MeV proton bombardment of an N_2_/H_2_ (10% H_2_ in N_2_) target using an Advanced Cyclotron Systems Inc. (Richmond, Canada) TR19 cyclotron. C-11 methane was converted to C-11 methyl iodide (^11^CH_3_I) in gas phase using a GE (Chicago, IL) TRACER FX C Pro module. Purification of ^11^C–MET was conducted using the HPLC component of the synthesis module on a Phenomenex (Torrance, CA) Aqua C18 semi-preparative column (5 μ, 250 × 10 mm). The HPLC solvent was phosphate-buffered saline (PBS, 3.93 mM) or PBS containing 100 ppm of ascorbate, and the flow rate was 3.0 mL/min. Millex-GS 0.22 μm sterile filter was purchased from EMD Millipore (Billerica, MA). Radioactivity was measured using a Capintec (Ramsey, NJ) CRC®-Ultra R dose calibrator. Mass analysis was performed using an AB SCIEX (Framingham, MA, USA) 4000 QTRAP mass spectrometer system with an ESI ion source.

### Synthesis and purification of ^11^C–MET

The tC18 Sep-Pak cartridge was preconditioned with EtOH (5 mL) and sterile water (10 mL). The remaining water in the cartridge was pushed out with air (10 mL). Five minutes before EOS, 85 μL of L-homocysteine thiolactone hydrochloride aqueous solution (25 mg in 600 μL water) was mixed with 200 μL of NaOH solution (0.7 mL of 10 N NaOH aqueous solution diluted with 4.3 mL water and 5.0 mL EtOH). From this, 200 μL of the mixed solution was loaded to the tC18 Sep-Pak cartridge. After passing ^11^CH_3_I by helium (15 mL/min) through the tC18 Sep-Pak cartridge, the reaction mixture was eluted off the cartridge with phosphate buffer (50 mM, 2 mL) and purified by HPLC. The eluate fraction (~ 1.5 mL) containing ^11^C–MET was collected, diluted with HPLC eluant (12 mL), and passed through a Millex-GS sterile filter into a final product vial.

### Quality control of ^11^C–MET

Chemical purity, radiochemical purity and radiochemical identity of ^11^C–MET and by-products were determined using an Agilent (Santa Clara, CA) HPLC system equipped with a model 1200 quaternary pump, a model 1200 UV absorbance detector (set at 220 nm), and a Bioscan (Washington, DC) NaI scintillation detector. The operation of the Agilent HPLC system was controlled using the Agilent ChemStation software. The HPLC column used was a Phenomenex Luna C18 analytical column (5 μ, 250 × 4.6 mm). The HPLC solvent was phosphate buffer (1 mM, pH 3), and the flow rate was 1.0 mL/min.

## Additional file

Additional file 1: Figure S1. Identification of METSO from decayed ^11^C–MET solution. (DOCX 260 kb)

## Abbreviations

^11^CH_3_I: Carbon-11 labeled methyl iodide
^11^C–MET: Carbon-11 labeled L-methionine
^11^C–METSO: Carbon-11 labeled methionine sulfoxide
EOS: End of synthesis
EtOH: Ethanol
HPLC: High performance liquid chromatography
NaOH: Sodium hydroxide
PBS: Phosphate-buffered saline
PET: Positron emission tomography
ppm: Part per million
*t*_R_: Retention time
UV: Ultraviolet light
