# Supplementary material for: Improving the stability of 11C–labeled L-methionine with ascorbate
Source: EJNMMI Radiopharm Chem. 2017 Oct 4;2:13. doi: 10.1186/s41181-017-0032-x (PMC5824703; doi:10.1186/s41181-017-0032-x)
Supplement: Supplementary file 1 — Identification of METSO from decayed 11C–MET solution. (DOCX 260 kb) [file 41181_2017_32_MOESM1_ESM.docx]

**Improving the stability of ^11^C-labeled L-methionine with ascorbate**

Michael Woods^1^, Leo Leung^1^, Kari Frantzen^1^, Jennifer G. Garrick^1^, Zhengxing Zhang^2^,

Chengcheng Zhang^2^, Wade English^1^, Don Wilson^1^, François Bénard^1,2,3^, Kuo-Shyan Lin^1,2,3*^

^1^Department of Functional Imaging, BC Cancer Agency, Vancouver, BC, Canada

^2^Department of Molecular Oncology, BC Cancer Agency, Vancouver, BC, Canada

^3^Department of Radiology, University of British Columbia, Vancouver, BC, Canada

**Corresponding author:**

Kuo-Shyan Lin, PhD

Address: 675 West 10^th^ Avenue, Rm 4-123, Vancouver, BC V5Z 1L3, Canada

Tel.: 1-604-675-8208; Fax: 1-604-675-8218; E-mail: [klin@bccrc.ca](mailto:klin@bccrc.ca)

**SUPPLEMENTAL INFORMATION**


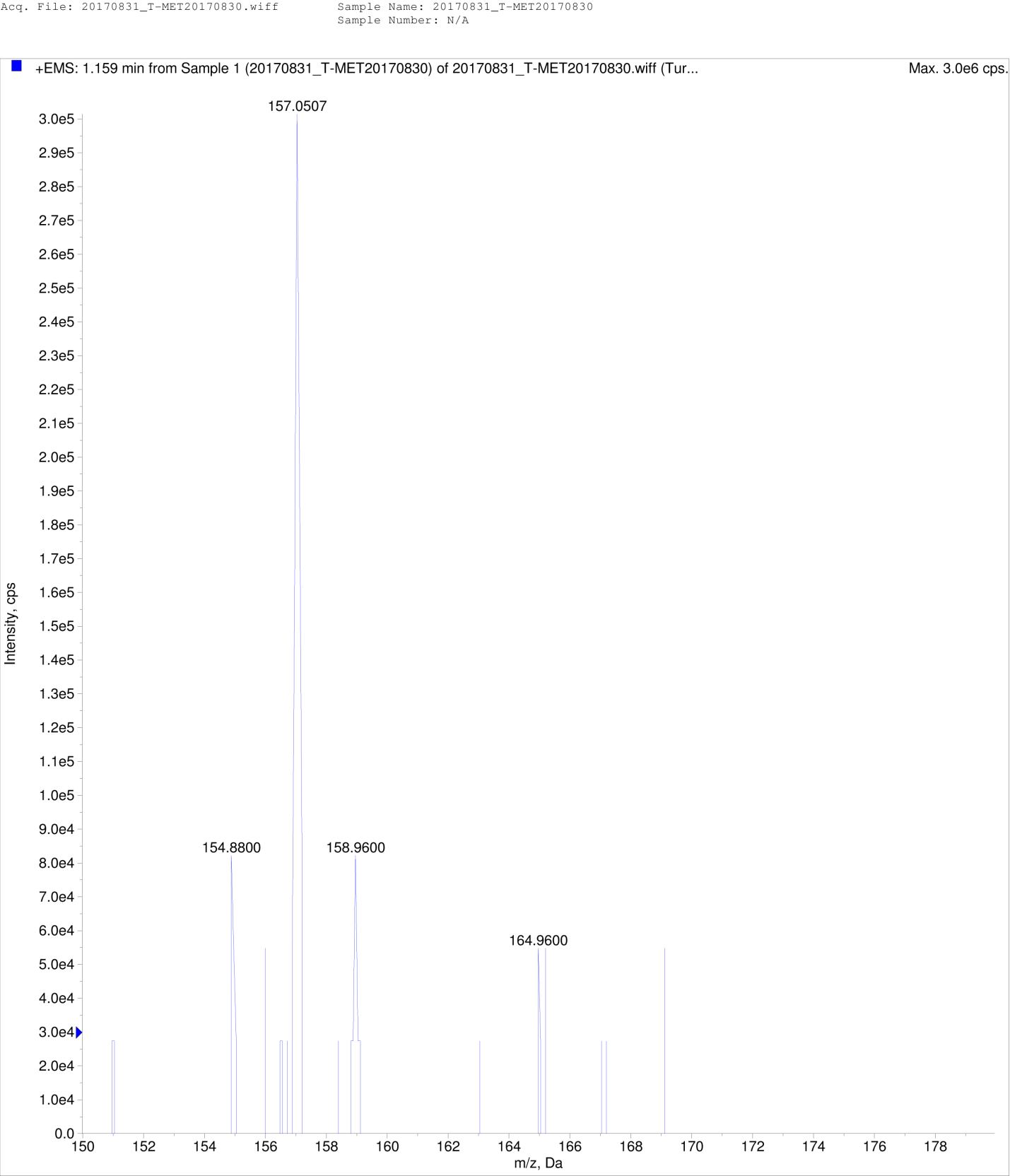


**Supplemental Figure 1**: Identification of METSO from decayed ^11^C-MET solution. Calculated mass of METSO C_5_H_11_NO_3_S 165.05, found [M]^+^ 164.96.
